# Identification and prognostic biomarkers among ZDHHC4/12/18/24, and APT2 in lung adenocarcinoma

**DOI:** 10.1038/s41598-024-51182-9

**Published:** 2024-01-04

**Authors:** Jing Bian, Wenji Xiong, Zhiguang Yang, Minzhe Li, Demei Song, Yanli Zhang, Chaoying Liu

**Affiliations:** 1 Department of Respiratory Medicine, The First Affiliated Hospital of Jilin University, Changchun, People’s Republic of China; 2 Department of Radiology, The First Hospital of Jilin University, Changchun, Jilin People’s Republic of China; 3 Department of Thoracic Surgery, The First Hospital of Jilin University, Changchun, 130021 People’s Republic of China; 4 Department of Respiratory and Critical Care Medicine, The First Hospital of Jilin University—The Eastern Division, Changchun, 130000 Jilin People’s Republic of China; 5 Central Laboratory, The First Hospital of Jilin University, Changchun, People’s Republic of China; 6 Key Laboratory of Organ Regeneration and Transplantation, Ministry of Education, Changchun, Jilin, 130021 People’s Republic of China; 7 Echocardiography Department, The First Hospital of Jilin University, Changchun, People’s Republic of China

**Keywords:** Cancer, Cancer microenvironment, Lung cancer

## Abstract

*S*-palmitoylases and *S*-depalmitoylases are differentially expressed in various cancers and several malignant tumors and show a strong prognostic ability. Notwithstanding, the potential clinical impact of *S*-palmitoylases and *S*-depalmitoylases, particularly in the prognosis and progression of lung adenocarcinoma (LUAD), has not been clarified. Expression levels of *S*-palmitoylases and *S*-depalmitoylases in LUAD were investigated using TCGA. GEPIA was used to evaluate the mRNA levels of *S*-palmitoylases and *S*-depalmitoylases at different pathological stages. Metascape was used to investigate the biological significance of *S*-palmitoylases and *S*-depalmitoylases. The Kaplan–Meier plotter was used to analyze the prognostic value of *S*-palmitoylases and *S*-depalmitoylases. CBioportal was used to analyze gene alterations in *S*-palmitoylases and *S*-depalmitoylases. UALCAN was used to examine DNA promoter methylation levels of *S*-palmitoylases and *S*-depalmitoylases. Finally, we investigated the relationship between *S*-palmitoylases, *S*-depalmitoylases, and tumor-infiltrating immune cells using TIMER. Correlations with immune checkpoint-related genes were determined using the R packages *reshape2*, *ggpubr, ggplot2,* and *corrplot*. PCR was also performed to assess the degree of *ZDHHC4/12/18/24* and *APT2* transcript expression in lung adenocarcinoma and adjacent normal lung tissues. HPA was utilized to investigate protein levels of *S*-palmitoylases and *S*-depalmitoylases in LUAD and normal lung tissue. Our study found that *ZDHHC2/3/4/5/6/7/9/12/13/16/18/20/21/23/24, APT1/2, PPT1, LYPLAL1, ABHD4/10/11/12/13* and *ABHD17C* mRNA expression was significantly upregulated in LUAD, whereas *ZDHHC1/8/11/11B/14/15/17/19/22, ABHD6/16A* and *ABHD17A* mRNA expression was significantly downregulated. The functions of the differentially expressed *S*-palmitoylases and *S*-depalmitoylases were mainly associated with protein-cysteine *S*-palmitoyltransferase and protein-cysteine *S*-acyltransferase activities. Patients with high expression of *ZDHHC4/12/18/24*, *APT2, ABHD4, ABHD11 and ABHD12* had a shorter overall survival. Infiltration of six immune cells (B cells, CD8^+^ T cells, CD4^+^ T cells, macrophages, neutrophils, and dendritic cells) was closely associated with the expression of *ZDHHC4/12/18/24* and *APT2*. *ZDHHC4/12/18/24* and *APT2* positively correlated with the immune checkpoint-related gene CD276. We assessed the mRNA levels of *ZDHHC4/12/18/24* and *APT2* using qRT-PCR and found increased expression of *ZDHHC4/12/18/24* in LUAD compared with healty control lung tissues. *ZDHHC4/12/18/24*, and *APT2* are potential prognostic biomarkers of LUAD. Their expression levels could be related to the tumor microenvironment in LUAD.

## Introduction

Statistical evidence from GLOBOCAN 2020 revealed that the second most prevalent cancer was lung cancer, which was the main cause of cancer-related mortality in 2020^1,2^. Of tumor types affecting the respiratory system, approximately 80–85 percent of all tumors are non-small cell lung cancers^3^. The most common subtypes of lung cancer include lung adenocarcinoma (LUAD) and lung squamous cell carcinoma (LUSC)^4^. LUSC is associated with undesirable clinical outcomes and the absence of efficient targeted treatment^5^. LUAD is the most prevalent subtype of lung cancer and has a poor prognosis^6^. The response of non-small cell carcinoma (NSCLC) to chemotherapy is not ideal, so surgery is the best treatment option; however, the curative effect of surgery is poor, except for limited tumors. Radiotherapy is effective in a few cases and palliative treatment in most cases. For advanced cases, chemotherapy can generally improve survival and alleviate symptoms. Therefore, it is crucial for the quick intervention and successful treatment of patients with NSCLC to make an early diagnosis of non-small cell lung cancer progression and to identify biomarkers.

*S*-palmitoylation is a reversible post-translational modification^7^. It is necessary for the trafficking and localization of regulatory proteins involved in cellular growth and signaling^8^. Protein depalmitoylation removes long-chain fatty acids which are linked to thioesters from cysteine residues in proteins. The *S*-depalmitoylases are comprised of *LYPLA1, LYPLA2, PPT1, LYPLAL1,ABHD4, ABHD6, ABHD10, ABHD12, ABHD13, ABHD16A, ABHD17A, ABHD17B, ABHD17C*^9^.

The catalytic core of ZDHHC protein acyltransferases (PATs) is located inside an invariant Asp-His-His-Cys (ZDHHC) cysteine-rich domain^10^. The human ZDHHC family comprises 23 enzymes (ZDHHC1-24*,* skipping ZDHHC10) that participate in the *S*-acylation of a variety of intracellular proteins, such as channels, receptors, transporters, and signaling molecules^11^. Functional changes in the ZDHHC family are related to multiple human disorders, including cancer and neurological conditions^12^. ZDHHC3 is overexpressed in breast cancer and high ZDHHC3 expression results in unfavorable survival of patients with breast cancer^13^. In addition, ZDHHC5 is elevated in NSCLC and has the potential to contribute to tumor formation^14^. This may imply that *S*-palmitoylases may be critical to the growth and metastasis of cancer, but the specific role of ZDHHCs in lung cancer still needs to be further studied. APT1 (acyl-protein thioesterase 1), also known as LYPLA1, catalyzes protein depalmitoylation^15^. Previous studies have demonstrated that blocking APT1 remarkably prevents migration, invasion, and population growth of NSCLC tumor cells, suggesting that APT1 acts as a tumor promoter in NSCLC cells in vitro^16^. Palmitoyl protein thioesterase 1 (PPT1) is a lysosomal enzyme that removes thioester-linked fatty acyl groups from cysteine residues within *S*-acylated proteins^17^. High PPT1 expression tends to decrease the chances of survival in individuals with several malignancies^18^. PPT1 is markedly upregulated in cancer and can effectively control tumor growth^19^.The ABHD17 family (including ABHD17A*,* ABHD17B*,* and ABHD17C), which belongs to the α/β-hydrolase folding superfamily^20^, catalyzes cancer-promoting N-Ras depalmitoylation and facilitates its repositioning to the endosomal membrane; accordingly, the ABHD17 family may impede the development of tumors^21^. However, the biological roles of the APT1/2, PPT1, and ABHD17 family members in lung cancer are not fully understood.

The expression pattern and predictive value of *S*-palmitoylases and *S*-depalmitoylases in LUAD have not yet been elucidated, and the identification of appropriate molecules as diagnostic indicators and therapeutic targets for lung adenocarcinoma remains an urgent problem.

In this investigation, we systematically studied the expression of mRNA, prognosis, and mutation of *S*-palmitoylases and *S*-depalmitoylases from a bioinformatic perspective. Importantly, we performed gene ontology and Kyoto Encyclopedia of Genes and Genomes analyses of *S*-palmitoylases and *S*-depalmitoylases. A comprehensive study may reveal novel therapeutic targets for the development of LUAD and help clinicians precisely predict patient prognosis.

## Materials and methods

### Data collection and data processing

The Cancer Genome Atlas (TCGA) is a publicly financed effort that seeks to identify and classify significant genetic abnormalities associated with cancer to produce an extensive “atlas” of cancer genomic profiles^22^. The clinical characteristic data of patients with LUAD were downloaded from the TCGA database, which included 535 LUAD samples and 59 adjacent normal tissues. The specific characteristics included age, sex, smoking status, survival status, TNM stage, and pathological stage (Table 1).

**Table1:** Characteristics of LUAD patients.

Characteristic	Levels	Overall
n		535
Sex, n (%)	Female	286 (53.5%)
	Male	249 (46.5%)
Pathologic stage, n (%)	Stage I	294 (55%)
	Stage II	123 (23%)
	Stage III	84 (15.7%)
	Stage IV	26 (4.9%)
	Unknown	8 (1.5%)
M stage, n (%)	M0	361 (67.5%)
	M1	25 (4.7%)
	MX	143 (26.7%)
	Unknown	6 (1.1%)
N stage, n (%)	N0	348 (65%)
	N1	95 (17.8%)
	N2	74 (13.8%)
	N3	2 (0.4%)
	NX	15 (2.8%)
	Unknown	1 (0.2%)
T stage, n (%)	T1	175 (32.7%)
	T2	289 (54%)
	T3	49 (9.2%)
	T4	19 (3.6%)
	TX	3 (0.6%)
Age, n (%)	> 65	261 (48.8%)
	≤ 65	255 (47.7%)
	Unknown	19 (3.6%)

Human specimens were obtained from the Department of Biobank, Division of Clinical Research, the First Hospital of Jilin University. Patients with LUAD who underwent surgery at the First Hospital of Jilin University participated in the study. Before surgery or cancer diagnosis, no patients had experience with chemotherapy, radiation, or antineoplastic therapy. Lung adenocarcinoma was histologically diagnosed in every patient. Lung cancer tissues were surgically removed, together with nearby normal tissues that were more than 5 cm distant from the tumor, and the tissues were stored at − 80 ℃. Age, gender, and TNM stage were among the clinical characteristics gathered. The AJCC Cancer Staging Manual, 8th Edition and the Veterans Administration Lung Cancer Study Group 3.30 criteria were used to establishing clinical stages. The Ethical Committee of the First Hospital of Jilin University approved our study. This study was conducted in accordance with the Declaration of Helsinki, and all patients provided written informed consent.

### Correlations with transcriptional levels of target genes

*S*-palmitoylases and *S*-depalmitoylases RNA-sequencing expression (level 3) profiles and associated clinical data were retrieved from the TCGA dataset (https://portal.gdc.com). The most recent version (V8) of the Genotype-Tissue Expression datas was downloaded from the Genotype-Tissue Expression project data portal website (https://www.gtexportal.org/home/datasets). We selected 513 lung adenocarcinoma samples from the TCGA database and 637 normal samples from the paracancerous and Genotype-Tissue Expression databases. Statistical analyses were performed using R software v4.0.3 (R Foundation for Statistical Computing, Vienna, Austria). Statistical significance was set at *p* < 0.05. The R package “pheatmap” was used for this process. The clinical data were also obtained from the TCGA dataset (https://portal.gdc.com). We performed two types of Cox regression analysis: univariate and multivariate. Using the 'forestplot' R package, the forest function was employed to compute the *p* value, hazard ratio, and 95% confidence intervals of each variable.

### The correlation between targets of gene expression and pathological stages

The web-based application called Gene Expression Profiling Interactive Analysis(GEPIA) uses TCGA and Genotype-Tissue Expression data to provide rapid and adjustable functionality. Differential expression analysis, profiling visualization, correlation analysis, and patient survival analysis are just a few of the essential interactive and adaptable features offered by Gene Expression Profiling Interactive Analysis^23^. In this investigation, we carried out analyses of the association between pathology stages and expression of *S*-palmitoylases and *S*-depalmitoylases using the “LUAD” datasets in Gene Expression Profiling Interactive Analysis. A *p* value was calculated using Student's *t*-test, and the threshold *p* value was set at 0.05.

### Creation of a protein–protein interaction network

STRING (http://string-db.org; version 11.0) was used to evaluate the functional relationships between nodes and characterize the protein co-regulation of *S*-palmitoylases and *S*-depalmitoylases^24^. The default threshold of 0.4 was used to assess whether an interaction was statistically significant. An extensive gene list annotation and analysis resource for experimental biologists is offered by the web-based portal Metascape^25^. Gene ontology (GO) analysis, including biological process, cellular component, and molecular function enrichment analysis, was conducted for the selected *S*-palmitoylases and *S*-depalmitoylases using Metascape, then. Statistical significance was defined as a *p* value < 0.05. ClueGO (version 1.5.3) was used to assess and depict the functional enrichment of gene ontology, including biological processes, cellular components, molecular function, and Kyoto Encyclopedia of Genes and Genomes (KEGG): BP, CC, MF, and KEGG.

### Survival analysis

The Kaplan–Meier plotter, an integrated database and online tool with multivariate and univariate analytic capabilities for in silico validation of novel biomarker candidates in NSCLC is available at www.kmplot.com/lung^26^. In this study, to assess the overall survival(OS), first-progression survival(FP)- time to first progression, and post-progression survival(PPS) which is time from progression to death, cancer patients were divided into high and low expression groups, based on the median levels of mRNA expression, which were then verified by K–M survival curves, hazard ratios with 95% confidence intervals, and log-rank *p* values. Statistical significance was set at *p* value < 0.05.

### Gene alterations and DNA methylation

The LUAD (TCGA, Firehose Legacy) dataset was selected for further analyses of *S*-palmitoylases and *S*-depalmitoylases, using the cBioPortal, an online tool for discovering, displaying, and analyzing multidimensional cancer genomics data for Cancer Genomics (http://cbioportal.org)^27^. We investigated the genetic profiles of *S*-palmitoylases and *S*-depalmitoylases in LUAD, which had mutations data and mRNA expression z-scores (RNASeq V2 RSEM) with a z-score cutoff of 1.8, and potential copy-number modifications from GISTIC software. We also analyzed the relationship between the mRNA expression of *ZDHHC4/12/18/24*, *APT2*, and methylation levels.

### Associations between DNA promoter methylation and transcript expression level

UALCAN, an interactive, user-friendly online platform^28^ was used to show the promoter methylation status of *ZDHHC4/12/18/24, APT2,ABHD4,ABHD11,and ABHD12* in LUAD and healthy tissues. The *p*-value was calculated using Student's *t*-test, and the threshold was set at 0.05.

### Immune infiltration analysis

The Tumor Immune Estimation Resource (cistrome.shinyapps.io/timer) is a platform for studying the connection between genes and tumor-infiltrating immune cells^29^. On the TIMER online website, we assessed the association between *S*-palmitoylases and *S*-depalmitoylases and B cells, CD4^+^ T cells, CD8^+^ T cells, dendritic cells, macrophages, and neutrophils.

### Correlation between *ZDHHC4/12/18/24* and *APT2* and immune checkpoint-related genes

An expression correlation analysis between *ZDHHC4/12/18/24* and *APT2* and immune checkpoint-related genes was performed. The ‘reshape2’, ‘ggpubr’, ‘ggplot2’, ‘ggpubr’, and ‘corrplot’ packages of R software were utilized in the procedure.

### Quantitative real-time polymerase chain reaction

TransZol (TransGen, CN) was used to collect the total RNA from the cells, and RNA concentration and purity were determined with BioTek Epoch 2 microplate Reader (Biotek, USA), after which cDNA was synthesized via reverse transcription using Hifair ® II 1st strand cDNA Synthesis SuperMix (Yeasen, CN). cDNA was used for subsequent quantitative real-time polymerase chain reaction (YEASEN, CN). A two-step process was performed using the 2XRealStar Green Fast Mixture with ROX II (GenStar, CN), with actin serving as the internal reference. The relative gene expression levels were calculated using the 2^–△△Ct^ method. The primer sequences are listed in the Supplementary Material (Supplementary 1. Table S1).

### Protein-level analysis of target genes

The Human Protein Atlas is a library of protein expression patterns based on immunohistochemistry in various cancers, normal tissues, and cell lines^30^. This database was used to acquire *S*-palmitoylase and *S*-depalmitoylase expression using immunohistochemistry images from clinical specimens taken from patients with LUAD compared with healthy control lung tissues.

### Predicted *S*-palmitoylation sites

GP* S*-Palm is a deep- learning-based graphic presentation system for the prediction of *S*-palmitoylation sites in proteins^31^. We speculated that CD276 may be palmitoylated by ZDHHC4/12/18/24 and affect tumor growth; therefore, we used this GP* S*-Palm software to predict CD276 palmitoylation sites.

### Statistical analysis

GraphPad Prism (version 7.0) was used to evaluate and present the transcript expression patterns in LUAD patients obtained from clinical samples. In both LUAD and adjacent healthy lung tissues, we assessed the mRNA expression levels of *ZDHHC4/12/18/24* and *APT2*. Differences were deemed significant at *p* < 0.05 following independent-sample Student’s* t*-test analysis.

## Results

### Aberrant expression of *S*-palmitoylases and *S*-depalmitoylases in patients with LUAD

**Figure 1:**
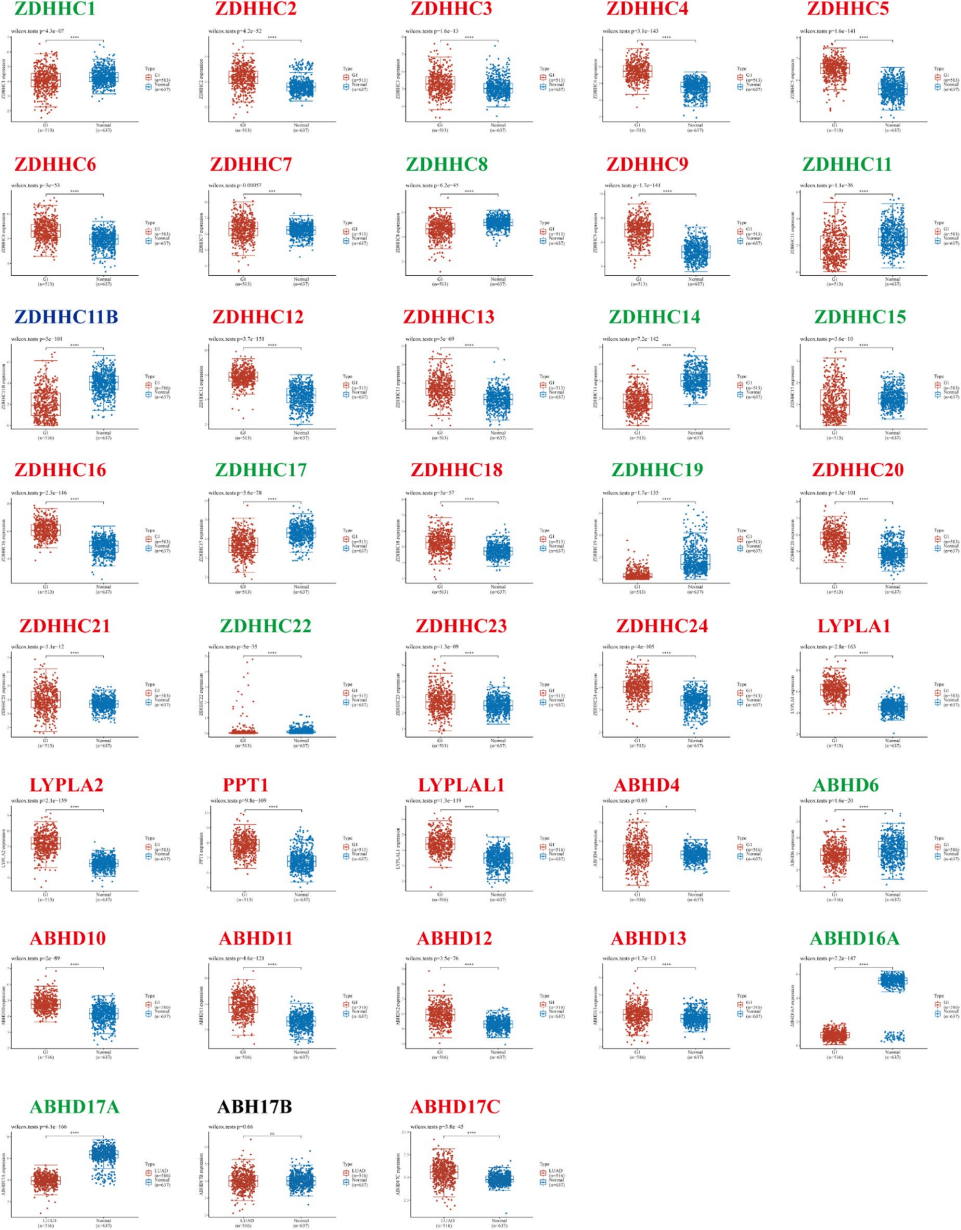
The transcriptional level of *S*-palmitoylases and *S*-depalmitoylases from the TCGA cohort. *S*-palmitoylases and *S*-depalmitoylases mRNA expression in LUAD tissues and normal tissues. The statistical differences between the two groups were determined by Student’s t-test and the p value cutoff was 0.05. The left red band stands for LUAD group and the right blue band for the healthy control group. ns = not significant; * *p* < 0.05, ** *p* < 0.01, *** *p* < 0.001.

The results showed that in LUAD, transcriptional levels of *ZDHHC2/3/4/5/6/7/9/12/13/16/18/20/21/23/24, APT1/2, PPT1, LYPLAL1, ABHD4/10/11/12/16A* and *ABHD17C* were upregulated, whereas *ZDHHC1/8/11/11B/14/15/17/19/22, ABHD6/16A* and *ABHD17A* were downregulated; the difference in expression of *ABHD17B* was not statistically significant (Fig. 1).

To identify whether *S*-palmitoylases and *S*-depalmitoylases were associated with tumorigenesis, progression, and clinical outcome, we investigated the link between the expression of each *S*-palmitoylase and *S*-depalmitoylase and the pathological stage of LUAD (Supplementary 2 Figure S1). The mRNA expression of *ZDHHC21* decreased as the tumor developed from stage I to stage II, suggesting a role for *ZDHHC21* in the control of LUAD carcinogenesis and development.

### Enrichment analysis of *S*-palmitoylases and *S*-depalmitoylases

**Figure 2:**
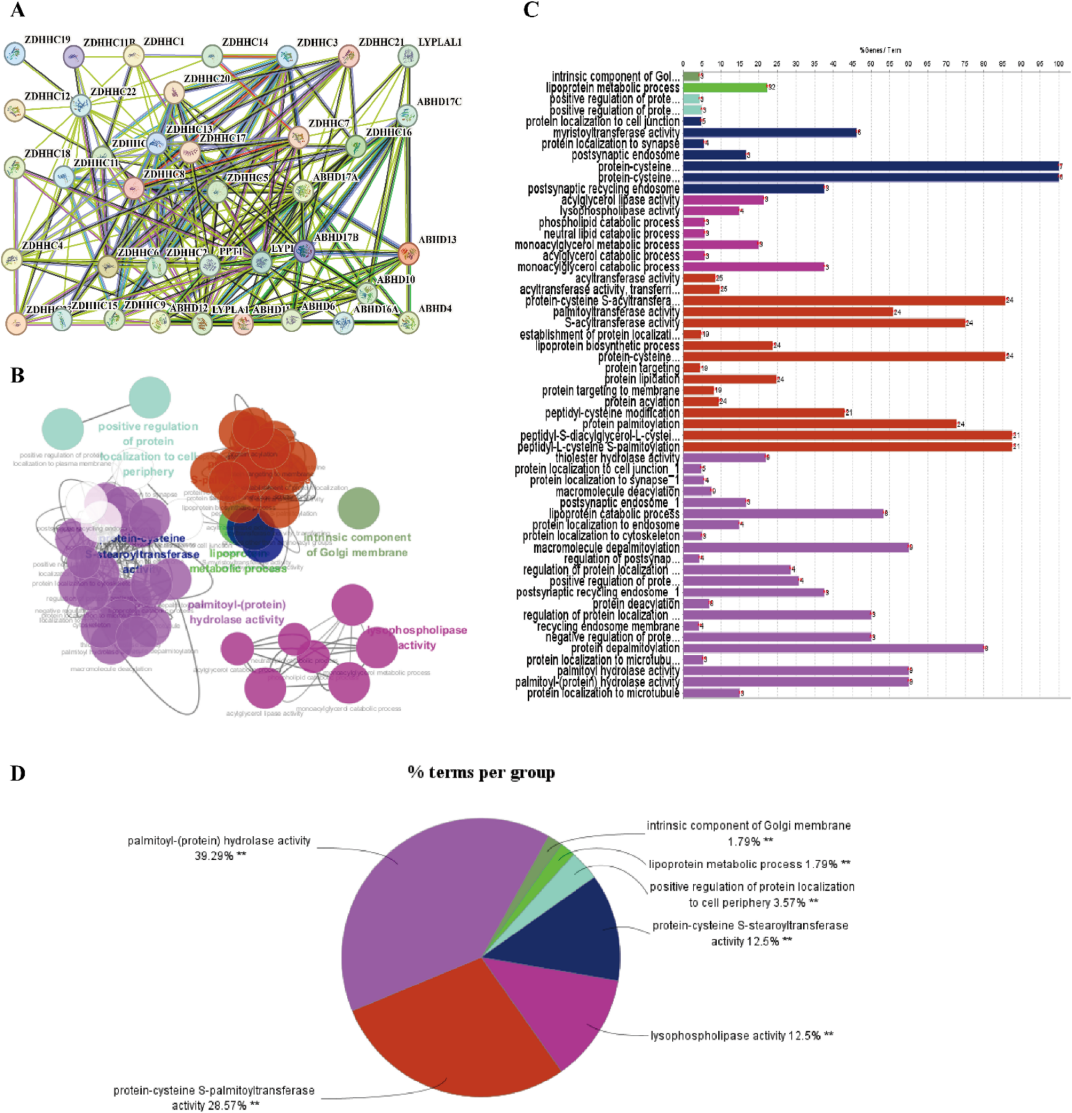
The enrichment analysis of *S*-palmitoylases and *S*-depalmitoylases in LUAD. (**A**) Protein–protein interaction network of *S*-palmitoylases and *S*-depalmitoylases. (**B**–**D**) The GO and KEGG functional annotation analysis of *S*-palmitoylases and *S*-depalmitoylases in LUAD was constructed using ClueGO, a plug-in of Cytoscape. The GO and KEGG enrichment analysis of the *S*-palmitoylases and *S*-depalmitoylases are shown in different colors. The results showed that significant differences in expression were related to protein-cysteine *S*-palmitoyltransferase activity and palmitoyl-(protein) hydrolase activity.

To investigate possible connections between differentially expressed *S*-palmitoylases and *S*-depalmitoylases, we used STRING to conduct protein–protein interaction network analysis. In line with our expectations, the protein–protein interaction network produced multiple nodes of 38 and several edges of 232 (Fig. 2A). Gene ontology analysis in METASCAPE was used to anticipate the roles of *S*-palmitoylases and *S*-depalmitoylases (Supplementary 3 Figure S2). Regarding cellular components, the intrinsic component of the Golgi membrane was most highly enriched. In the molecular function category, protein-cysteine *S*-palmitoyltransferase, protein-cysteine *S*-stearoyl transferase, and palmitoyl-(protein) hydrolase activities were enriched. In the biological processes category, lipoprotein metabolic processes, regulation of protein localization to the plasma membrane, and fatty acid derivative metabolic processes were significantly enriched. Functional annotations from ClueGO and CluePedia indicated a network of *S*-palmitoylases and *S*-depalmitoylases (Fig. 2B and 2C). The list of comprehensive functional notes and categorization pie charts were shown in Fig. 2D: 28.57% of terms belong to protein-cysteine *S*-palmitoyltransferase activity, 39.29% to palmitoyl-(protein) hydrolase activity, 12.5% to protein-cysteine *S*-stearoyltransferase activity, 1.79% to intrinsic component of Golgi membrane, 3.57% to positive regulation of protein localization to cell periphery, and 1.79% to lipoprotein metabolic process.

### Prognostic value of *S*-palmitoylases and *S*-depalmitoylases in LUAD

To evaluate whether the differential expression of *S*-palmitoylases and *S*-depalmitoylases play a role in LUAD development, we evaluated *S*-palmitoylases and *S*-depalmitoylases that were differentially expressed and notably linked with clinical outcomes in LUAD.

**Figure 3:**
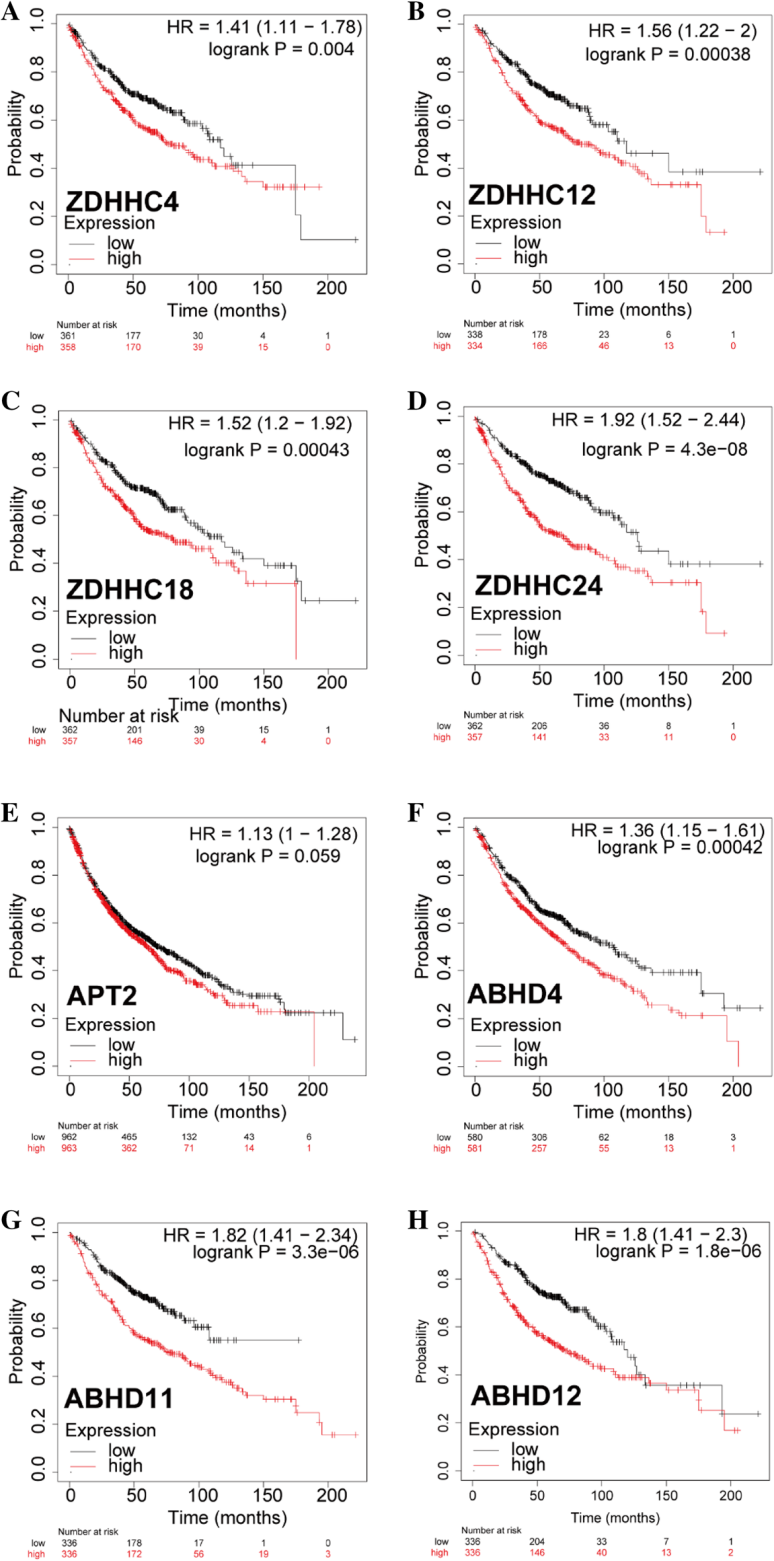
Overall survival analysis of *ZDHHC4/12/18/24*, *APT2, ABHD4, ABHD11, and ABHD12* in LUAD patients. Patients were divided into two groups including the high (the red line) and low (the black line) groups expression based on *ZDHHC4/12/18/24*, *APT2, ABHD4,ABHD11, and ABHD12* expression level. A log-rank test was used to estimate the difference in overall survival and a value of *p* < 0.05 was considered statistically significant.

Using Kaplan–Meier plotter datasets, we found that patients with high transcriptional levels of *ZDHHC4/12/18/24, APT2, and ABHD4/11/12* had shorter overall survival times than those with low levels(Fig. 3). Patients with LUAD with a high transcriptional expression of *ZDHHC2/3/6/9/11/13/14 /15 /16 /17/20/21, APT1,* and *ABHD17B* tended to have better overall survival (Supplementary 4 Figure S3). High expression of *ZDHHC3/6/7/16/20/21, ATP1, LYPLAL1,ABHD13, and ABHD17C* were associated with better first progression survival (Supplementary 5 Figure S4). High expression of *ZDHHC12/24, and ABHD4/11/12* tended to result in worse first-progression survival. A better post-progression survival was associated with higher *ZDHHC3/6/13/20/21, LYPLAL1 and ABHD13* expression levels (Supplementary 6 Figure S5).

### Correlations between clinical characteristics and *ZDHHC4/12/18/24* and* APT2* transcriptional expression in LUAD

Patients were separated into two groups based on the median level of *ZDHHC4/12/18/24* and *APT2* transcript expression in the TCGA database: a high expression group and a low expression group. High *ZDHHC4* mRNA expression was related to smoking (*p* = 0.001) and lymph node metastasis (*p* = 0.021) (Supplementary 7. Table S2). High *ZDHHC12* transcript expression was associated with lymph node metastasis (*p* = 0.021) and pathological stage (*p* = 0.024) (Supplementary 8. Table S3). In addition, high *ZDHHC18* transcript expression was associated with lymph node metastasis (*p* < 0.001) and pathological stage (*p* = 0.003) (Supplementary 9. Table S4). High *ZDHHC24* transcript expression was associated with sex (*p* = 0.034), lymph node metastasis (*p* = 0.02), and pathological stage (*p* = 0.008) (Supplementary 10. Table S5). High *LYPLA2* transcript expression was associated with sex (*p* = 0.034) (Supplementary 11 Table S6).

### Gene mutation of *S*-palmitoylases and *S*-depalmitoylases in patients with LUAD and promoter methylation of *ZDHHC4/12/18/24* and* APT2*

**Figure 4:**
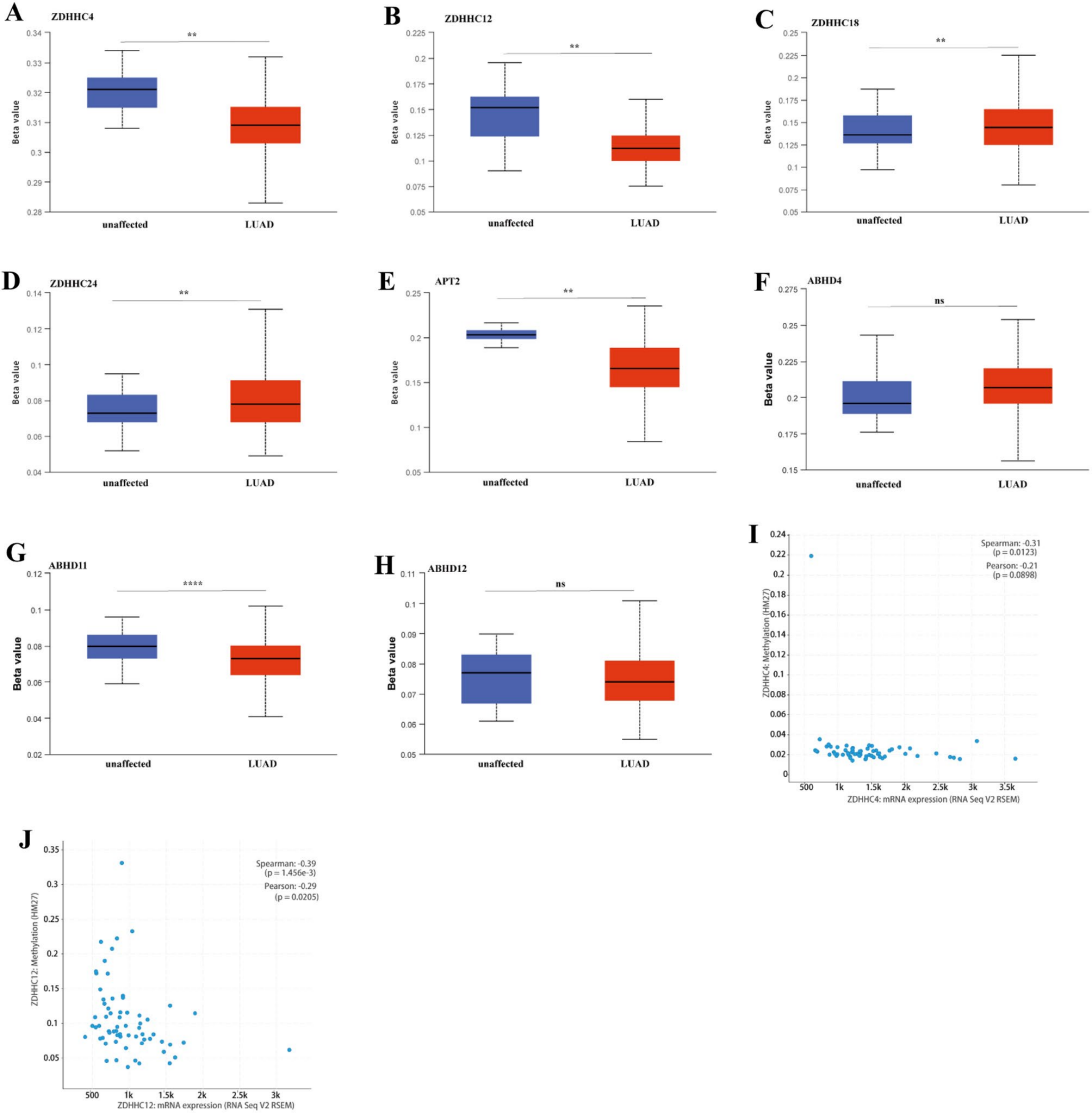
Decreased DNA methylation may contribute to increased expression of *ZDHHC4/12* in LUAD. (**A**–**H**) The DNA methylation level of *ZDHHC4/12/18/24*, *APT2, ABHD4, ABHD11, and ABHD12* in LUAD tissues. The Beta value indicates level of DNA methylation ranging from 0 (unmethylated) to 1 (fully methylated). (**I**, **J**) The correlation of *ZDHHC4/12* DNA methylation level and ZDHHC4/12 mRNA expression level.

Next, we examined the molecular properties of *S*-palmitoylases and *S*-depalmitoylases in LUAD. Gene alterations were analyzed using cBioportal. The results showed that *ZDHHC1/2/3/4/5/6/7/8/9/11/11B/12/13/14/15/16/17/18/19/20/21/22/23/24, APT1/2, PPT1*, *ABHD4, ABHD6, ABHD10,ABHD11,ABHD12,ABHD13,ABHD16A*and *ABHD17A/B/C* were altered in 0.9%, 7%, 0.9%, 5%, 0.9%, 2.6%, 2.2%, 3%, 2.6%, 18%, 17%, 1.3%, 0.9%, 5%, 3%, 1.7%, 2.6%, 1.3%, 3%, 2.2%, 3%, 1.3%, 1.3%, 1.7%, 4%, 1.7%, 1.7%, 7%,1.7%, 0.4%,2.2%,0.9%,1.7%,1.3%,5%,1.3%,0.4% and 1.3% of LUAD samples, respectively. Amplification was the most common change observed in the samples (Supplementary 12 Figure S6). Factors that increase *S*-palmitoylases and *S*-depalmitoylases are not known, so we hypothesized that a correlation may exist between *S*-palmitoylase and *S*-depalmitoylase expression and methylation. We found that compared with normal lung tissue, promoter methylation levels of *ZDHHC4/12*, *APT2* and *ABHD11* were decreased in LUAD, whereas the promoter methylation level of *ZDHHC18/24* was increased (Fig. 4A). Additionally, cBioportal was used to confirm the connection between the methylation level and *ZDHHC4/12/18/24*, *APT2*, *ABHD4, ABHD11,*and *ABHD12* transcriptional levels. *ZDHHC18/24*, *APT2, ABHD4, ABHD11*, and *ABHD12* methylation data were not present in cBioportal. *ZDHHC4/12* methylation and expression levels in LUAD were negatively correlated (Fig. 4B).

### Relationship between immune cell infiltration and *S*-palmitoylase and *S*-depalmitoylase expression in patients with LUAD

**Figure 5:**
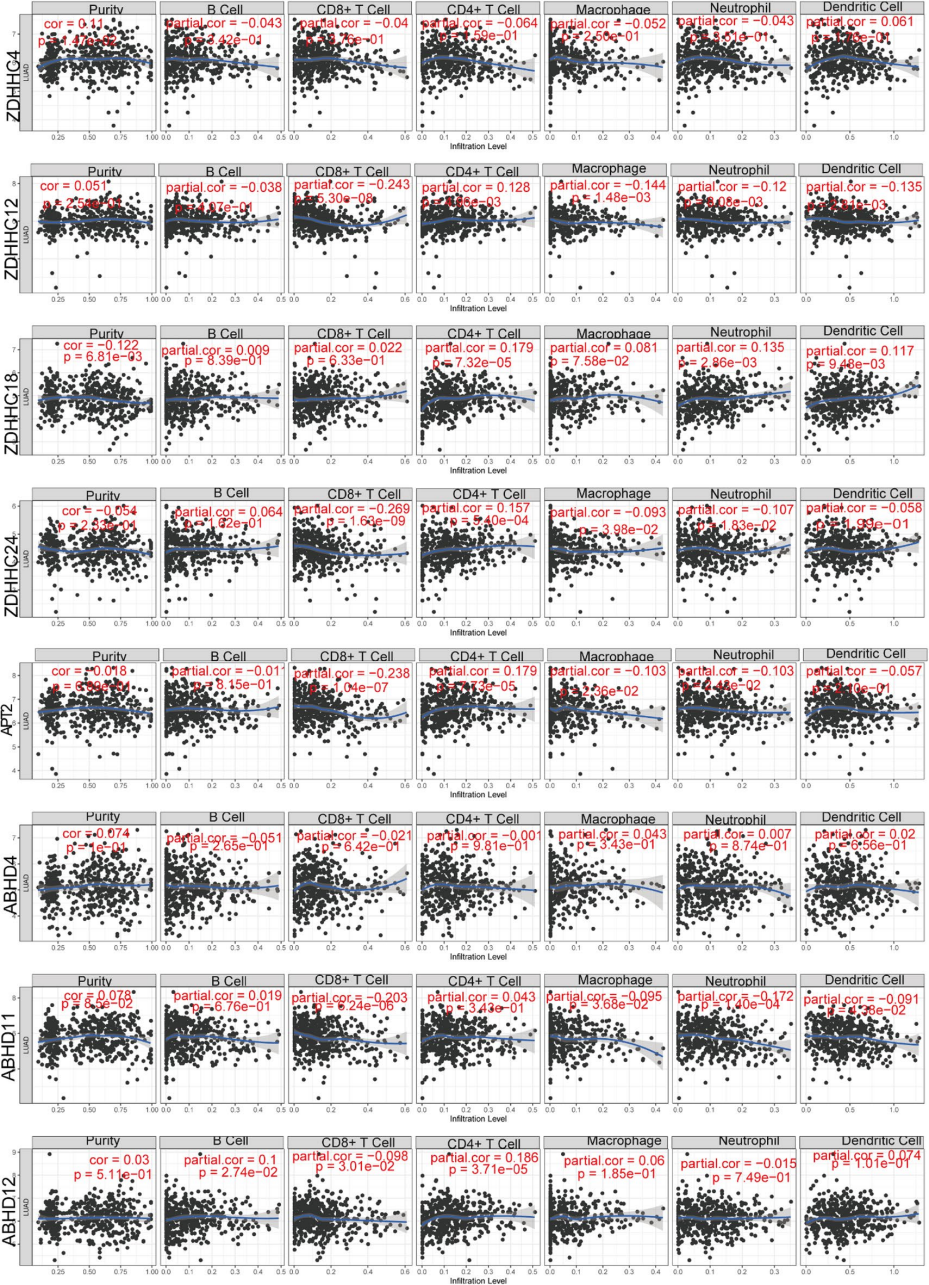
Correlation of *ZDHHC4/12/18/24*, *APT2,ABHD4,ABHD11,and ABHD12* expression in LUAD with infiltration levels of purity, B cell, CD8^+^ T cells, CD4^+^ T cell, macrophages, neutrophils, and dendritic cells. Spearman correlation coefficient was chosen for the correlation analysis and a value of *p* < 0.05 was considered statistically significant.

To investigate whether *S*-palmitoylases and *S*-depalmitoylases are effective biomarkers for immune therapy, we investigated the relationship between expression of *S*-palmitoylases and *S*-depalmitoylases and immune infiltration levels in LUAD. *ZDHHC4* expression negatively correlated with B cells, CD8^+^ T cells, CD4^+^ T cells, macrophages, and neutrophils. *ZDHHC12* expression was negatively associated with B cells, CD8^+^ T cells, macrophages, neutrophils, or dendritic cells. In addition, *ZDHHC18* expression positively correlated with B cells, CD8^+^ T cells, CD4^+^ T cells, macrophages, neutrophils, and dendritic cells. *ZDHHC24* expression was positively correlated with B cells and CD4^+^ T cells and negatively correlated with CD8^+^ T cells. *APT2* expression negatively correlated with B cells, CD8^+^ T cells, CD4^+^ T cells, macrophages, neutrophils, and dendritic cells. And *ABHD11* expression was negatively correlated with CD8^+^ T cells. However, *ABHD4* and *ABHD12* were not correlated with immune cell infiltration in LUAD. (Fig. 5).

### Correlation between *ZDHHC4/12/18/24* and *APT2* expression and immune checkpoint-related genes

**Figure 6:**
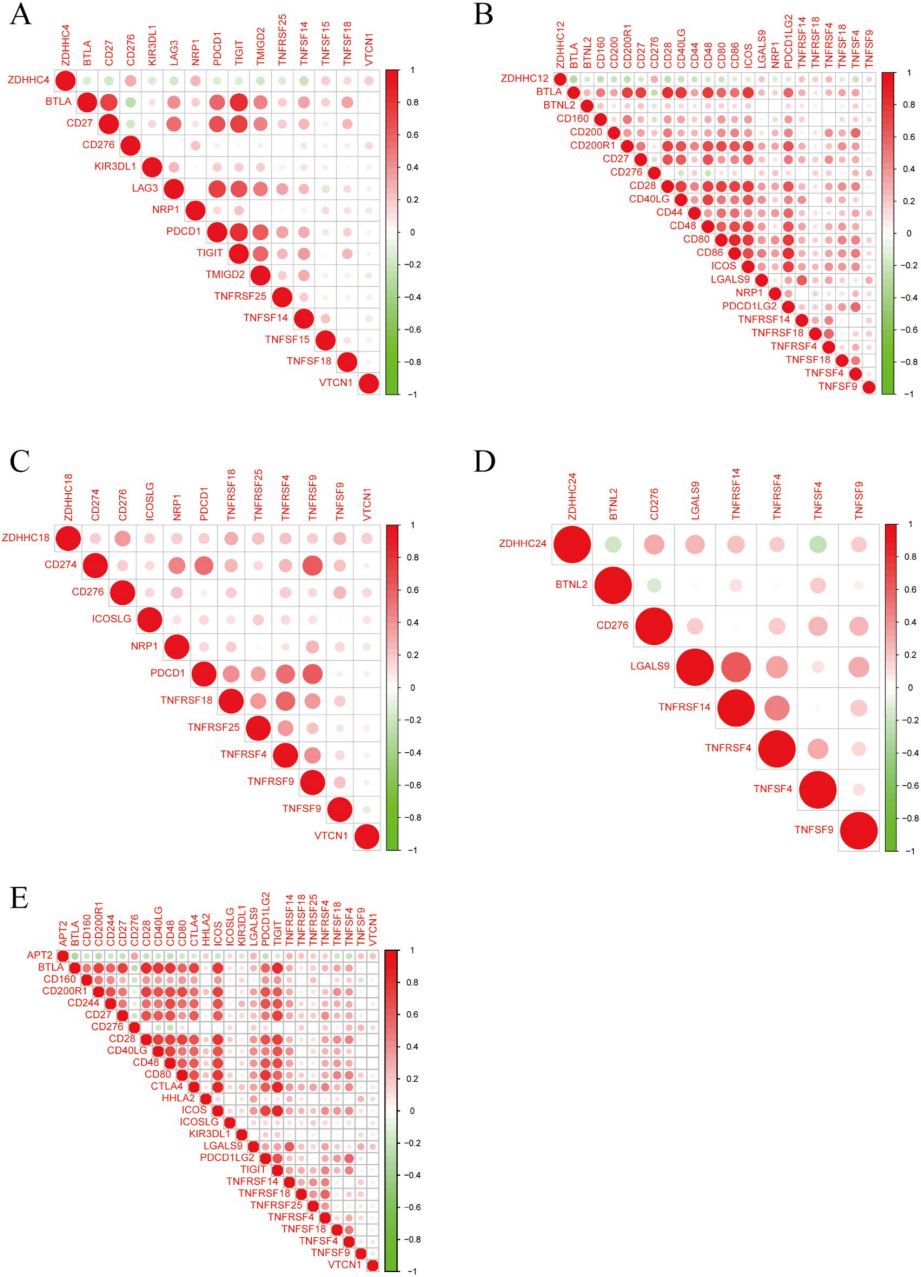
Correlation between *ZDHHC4/12/18/24* and *APT2* and immune checkpoint-related genes.

Next, we explored the correlation between the expression of *ZDHHC4/12/18/24, APT2*, and immune checkpoint-related genes. *ZDHHC4* expression was positively associated with *CD276, NRP1, TNFSF15, TNFSF18*, and *VTCN1* and negatively associated with *BTLA, CD27, KIR3DL1, LAG3, PDCD1, TIGIT, TMGD2*, and *TNFSF14. ZDHHC12* expression was positively associated with *CD276, LGALS9, PDCD1LG2*, and *TNFRSF4* and negatively associated with *BTLA, CD160*, and *CD200R1. ZDHHC18* expression positively correlated with *CD276, TNFRSF18*, and *TNFSF9. ZDHHC24* expression positively correlated with *CD276, LGALS9, TNFRSF14, TNFRSF4*, and *TNFSF9* and negatively correlated with *BTNL2* and *TNFSF4*. *APT2* expression positively correlated with *CD276, HHLA2,* and *LGALS9* and negatively correlated with *BTLA, CD48*, and *PDCD1LG2* (Fig. 6).

### *ZDHHC4/12/18/24* expression is considerably higher in lung adenocarcinoma tissues

**Figure 7:**
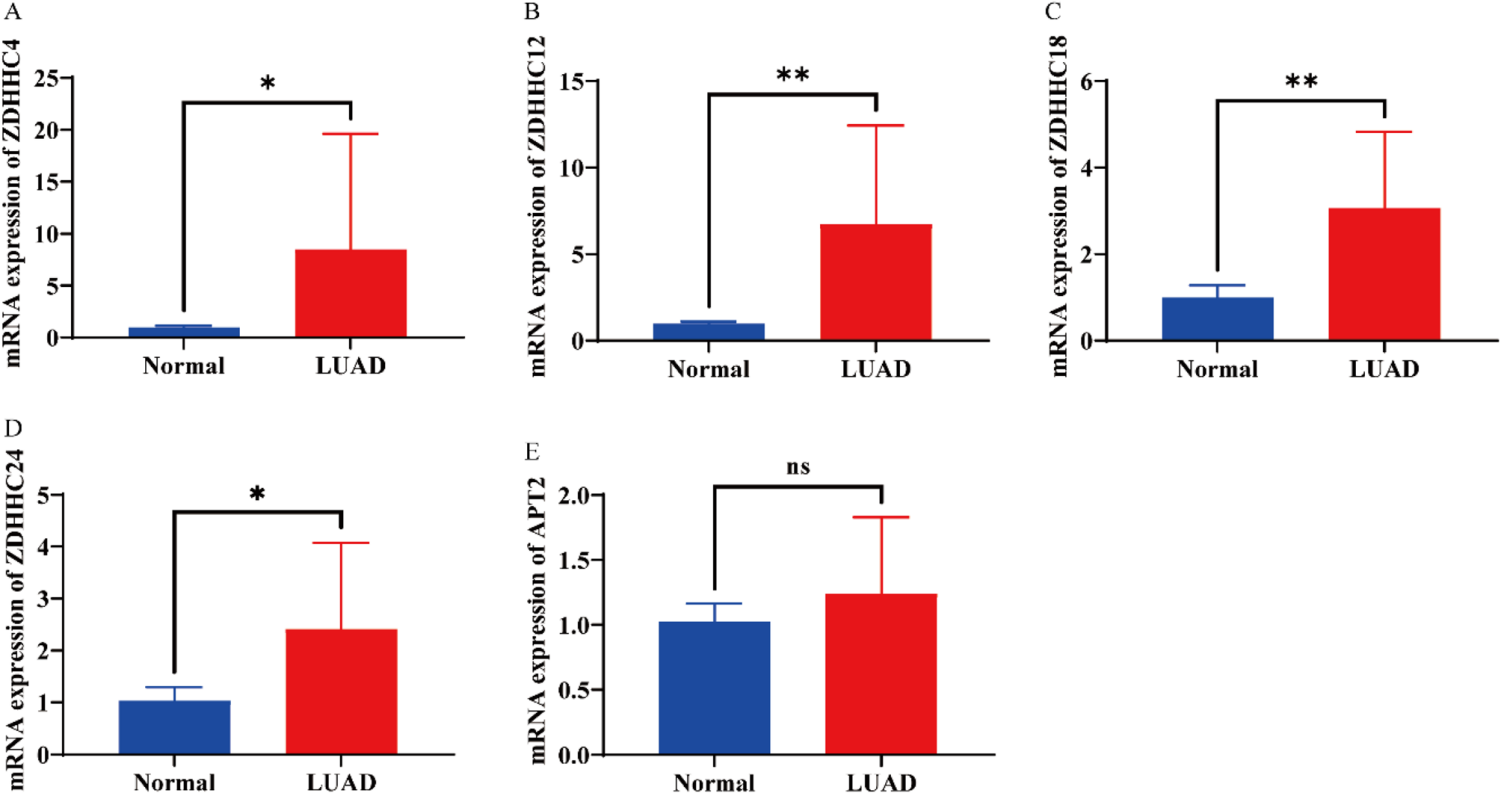
Increased expression of *ZDHHC4/12/18/24* in LUAD cancer tissues. *ZDHHC4* mRNA expression in LUAD tissues and corresponding adjacent tissues. (**A**) *ZDHHC12* mRNA expression in LUAD tissues and corresponding adjacent tissues. (**B**) *ZDHHC18* mRNA expression in LUAD tissues and corresponding adjacent tissues. (**C**) *ZDHHC24* mRNA expression in LUAD tissues and corresponding adjacent tissues. (**D**) *APT2* mRNA expression in LUAD tissues and corresponding adjacent tissues. (**E**) Differences were deemed significant at *p* < 0.05 following independent-sample Student’s t-test analysis. *p < 0.05; **p < 0.01; **** p < 0.001.

We found that the upregulation of *ZDHHC4/12/18/24* and *APT2* in LUAD and the high expression of *ZDHHC4/12/18/24* and *APT2* correlated with undesirable clinical consequences. Therefore, we intended to authenticate the mRNA expression levels of *ZDHHC4/12/18/24* and *APT2* in LUAD and compare them with normal lung tissues in vitro. We conducted a quantitative real-time polymerase chain reaction to compare the mRNA expression levels of *ZDHHC4/12/18/24* and *APT2* in LUAD and found that *ZDHHC4/12/18/24* expression was significantly upregulated in LUAD. The *APT2* expression level was not significantly different between LUAD and normal lung tissues (Fig. 7).

## Discussion

The ZDHHC protein family contains a conserved protein domain consisting of 51 amino acids, and the domain contains a motif that is embedded in a cysteine-rich domain^32^. Recent genetic analysis in yeast showed that ZDHHC proteins mediate palmitoylation^33^. Most members of the *ZDHHC* family are associated with many human diseases such as neuropsychiatric diseases and cancers^34^. The function of the *ZDHHC* family in cancer has been the subject of extensive research. For instance, several different types of cancers have been shown to have excessive *ZDHHC3* expression, such as breast cancer^13^. Over the past decades, studies have demonstrated that *ZDHHC8* siRNA and X-irradiation significantly slowed tumor development in 211H tumor-bearing mice^35^. Furthermore, *ZDHHC9* has also been reported to be overexpressed in diverse types of cancers. Disruption of PD-L1 palmitoylation by preventing *ZDHHC9* expression makes breast cancer cells more susceptible to T-cell-initiated death, which inhibits tumor development^36^. In previous reports, it was found that *ZDHHC11* is overexpressed in NSCLC and high-grade bladder cancers^37,38^. In addition, we found that ZDHHC12 participated in apoptosis. Additionally, the BCL-2 associated X protein BAX, which is an essential component of the intrinsic apoptotic pathway of cell apoptosis^39^, maybe palmitoylated more frequently as a result of ZDHHC12 overexpression. Therefore, we hypothesize that ZDHHC12 might modify BAX palmitoylation to control tumor cell death, thereby affecting tumor cell proliferation. In addition, ZDHHC16 has good application prospects for improving the prognosis of hepatocellular carcinoma patients^40^. In gliomas, *ZDHHC18* was upregulated and was associated with malignancy^41^. Previous studies have demonstrated that *PPT1* is increased in cancer and is crucial for constraining tumor growth^18^. However, the expression of all *S*-palmitoylases and *S*-depalmitoylases in LUAD had not yet been comprehensively investigated. Therefore, a better understanding of the function of *S*-palmitoylases and *S*-depalmitoylases in LUAD was necessary for the future development of *S*-palmitoylases and *S*-depalmitoylases in LUAD therapy.

In the current study, we investigated the expression and prognostic value of mutations and immune cell infiltration of *S*-palmitoylases and *S*-depalmitoylases in LUAD and their corresponding normal tissues. We screened the expression of *S*-palmitoylases and *S*-depalmitoylases with TCGA datasets. *ZDHHC2/3/4/5/6/7/9/12/13/16/18/20/21/23/24, APT1/2, PPT1, LYPLAL1, ABHD4/10/11/12/16A* and *ABHD17C* transcriptional level were upregulated in LUAD, whereas *ZDHHC1/8/11/14/15/17/19/22, ABHD6/13* and *ABHD17A* transcripts were downregulated. We then utilized GO and KEGG pathway enrichment analysis to examine the roles of genes connected to *S*-palmitoylases and *S*-depalmitoylases. The functions of *S*-palmitoylases and *S*-depalmitoylases mainly included protein palmitoylation, protein depalmitoylation, and acetylation.

We also conducted quantitative real-time polymerase chain reaction assays to compare transcript expression levels of *ZDHHC4/12/18/24* and *APT2* in LUAD and found that *ZDHHC4/12/18/24* expression was in accordance with TCGA analysis results. However, we may not have obtained exact findings because of the small sample size. Furthermore, the specimens collected from patients may exist in different pathological stages. We verified the expression of *S*-palmitoylases and *S*-depalmitoylases at the protein level, using the Human Protein Atlas database. We discovered that ZDHHC4/18/24 expression was noticeably higher in LUAD tissues than in normal tissues, however, ABHD4/11/12 protein level were decreased than in LUAD tissues (Supplementary 13 Figure S7).

In previous studies, differentially expressed *S*-palmitoylases and *S*-depalmitoylases have been found to affect patient survival. For example, we found that higher *ZDHHC3* expression correlated with decreased survival in patients with breast cancer^13^. Based on the results of this study, we found a strong correlation between human *ZDHHC8* expression and cancer survival^42^. Furthermore, genetic inhibition of *S*-depalmitoylase *PPT1* suppresses tumor growth, and high *PPT1* expression tends to aggravate the survival of patients with numerous distinct cancers^18^. In our study, according to the results from the Kaplan–Meier plotter, patients with LUAD with high transcript levels of *ZDHHC4/12/18/24* and *APT2* had a shorter overall survival time than those with low levels. These data demonstrate that *ZDHHC4/12/18/24* and *APT2* in LUAD may predict survival outcomes. Moreover, numerous malignancies have been found to depend heavily on *ZDHHC12* expression; for example, knockdown of *ZDHHC12* inhibits the malignant CLDN3 accurate membrane localization and tumorigenesis of ovarian cancer^39^. In glioblastoma, knockdown of *ZDHHC12* restrains the growth, migration, and invasion capabilities^43^. In our study, poor overall survival in LUAD was linked to high expression of *APT2*. Therefore, *APT2* expression may be an indicator of the prognosis of LUAD. As previously reported, *APT1* and *APT2* are significantly overexpressed in chronic lymphocytic leukemia cells. *APT*s directly interact with CD95 to accelerate depalmitoylation, which damages CD95-mediated apoptosis. CD95-mediated apoptosis in chronic lymphocytic leukemia cells can be reversed by treatment with miR* S*-138/-424^44^. Another study suggested that in Snail-transformed cells, blockade of *APT2* recovers Scribble localization and *S*-palmitoylation^45^. Therefore, *ZDHHC4/12/18/24* and *APT2* may serve as biomarkers for predicting survival in patients with LUAD.

Since *S*-palmitoylases and *S*-depalmitoylases varied markedly in LUAD, we examined the molecular features of LUAD. The main results of *S*-palmitoylases and *S*-depalmitoylases mutation were increased mRNA transcript expression. The mutation rates of all *S*-palmitoylases and *S*-depalmitoylases were low. On investigating the relationship between *S*-palmitoylase and *S*-depalmitoylase transcript expression levels and promoter methylation in LUAD, we found that the differentially expressed *ZDHHC4/12/18/24* and *APT2* were influenced by methylation levels, and expression levels of *ZDHHC4/12* and *APT2* were negatively correlated with the methylation level. These findings support earlier research that found a negative correlation between *ZDHHC12* methylation and expression in glioblastomas^43^.

Immune cell infiltration plays a significant role in tumor progression and prognosis^46^. In this study, we found a significant correlation between the expression of ZDHHC4/12/18/24 and APT2 and the infiltration of the immune cells, suggesting that ZDHHC4/12/18/24 and APT2s are not only as prognostic indicators, but may also reflect immune status. In general, tumor-infiltrating cells, especially CD8^+^ T cells, have a good prognosis for NSCLC^47^. In LUAD, the expression of *ZDHHC12/24* and *APT2* was negatively correlated with CD8^+^ T cells. This suggests that *ZDHHC12/24* and *APT2* may inhibit tumor-associated CD8^+^ T cells, thereby aggravating the pathogenesis of LUAD and promoting tumor growth. We also investigated the correlation between immune checkpoint-related genes and the expression of *ZDHHC4/12/18/24* and *APT2.* There was an evident positive association between the expression of the immune checkpoint-related gene CD276 and the expression of *ZDHHC4/12/18/24* and *APT2*. The immune checkpoint-related gene, CD276, is commonly known as B7-H3. Previous research has shown that CD276 helps tumor cells evade the immune system in non-small cell lung cancer by collaborating with Tregs^48^. From several previous study, we had determined that CD276 might affect tumors via the JAK-STAT3 pathway^49–51^. In this study, GP* S*-Palm software identified three high-scoring palmitoylation sites in CD276 (Supplementary 14 Table S7). Therefore, we propose that CD276 might be palmitoylated, which would impact protein function. In this study, we demonstrated that *ZDHHC4/12/18/24* and *APT2* may affect CD276 palmitoylation and, therefore, tumor growth.

Our study has certain limitations. First, despite the high expression of *ZDHHC4/12/18/24* and *APT2* in LUAD as a strong prognostic factor for the survival of LUAD, almost all data used in our investigation were obtained from a web database. Therefore, further studies are required to confirm our findings and investigate the therapeutic use of *S*-palmitoylases and *S*-depalmitoylases in cancer treatment. Second, we did not explore the mechanisms of *S*-palmitoylases and *S*-depalmitoylases in LUAD. Future studies should explore these underlying mechanisms. Furthermore, *ZDHHC4/12/18/24* and *APT2* play crucial roles in the onset and progression of LUAD. These markers also have prognostic and diagnostic relevance. However, while this exhibits potential value, it is also uncertain, and further verification of the outcomes of los* S*-of-function and gain-of-function is required.

## Conclusion

This study found that *ZDHHC2/3/4/5/6/7/9/12/13/16/18/20/21/23/24, APT1/2, PPT1, LYPLAL1, ABHD4, ABHD10, ABHD11,ABHD12,ABHD13* and *ABHD17C* mRNA expression was significantly upregulated in LUAD, whereas *ZDHHC1/8/11/11B/14/15/17/19/22, ABHD6,ABHD16A* and *ABHD17A* mRNA expression was significantly downregulated. The functions of the differentially expressed *S*-palmitoylases and *S*-depalmitoylases were mainly associated with protein-cysteine *S*-palmitoyltransferase and palmitoyl-(protein) hydrolase activities. Patients with high expression of *ZDHHC4/12/18/24* and *APT2* had a shorter overall survival. Infiltration of six immune cells (B cells, CD8^+^ T cells, CD4^+^ T cells, macrophages, neutrophils, and dendritic cells) was closely associated with the expression of *ZDHHC4/12/18/24* and *APT2*. *ZDHHC4/12/18/24* and *APT2* positively correlated with the immune checkpoint-related gene CD276. We assessed the mRNA levels of *ZDHHC4/12/18/24* and *APT2* using quantitative real-time polymerase chain reaction and found increased expression of *ZDHHC4/12/18/24* in LUAD compared with normal lung tissues.

### Supplementary Information

Supplementary Information.

## Data Availability

The data of this manuscript are all presented in the article.
