# Supplementary material for: Identification and prognostic biomarkers among ZDHHC4/12/18/24, and APT2 in lung adenocarcinoma
Source: Sci Rep. 2024 Jan 4;14:522. doi: 10.1038/s41598-024-51182-9 (PMC10767092; doi:10.1038/s41598-024-51182-9)
Supplement: Supplementary file 1 — Supplementary Information. [file 41598_2024_51182_MOESM1_ESM.docx]

**Supplementary 1** **TABLE 1**: Sequences of the primers.

| **Gene** | **Former primes** | **Reverse primers** |
| --- | --- | --- |
| *ZDHHC4* | 5'- GTACACCTGGGAAGTATTTGGC -3' | 5'- CTAGCAGCAGATAGGGCAGAA -3' |
| *ZDHHC12* | 5'- CTCCTCGTCTCGCACCTCTA -3' | 5'- GATAGGCGATGCGGTGTGAG -3' |
| *ZDHHC18* | 5'- CACCCCGAACCTCACACTG T-3' | 5'- TGAAGGCCGTCAGGAATGAGA-3' |
| *ZDHHC24* | 5'- CTGGCACAGTTTGCCTTGG T-3' | 5'- CAGGGACCCAGGTCATAGGAG-3' |
| *APT2* | 5'- CCCTCACGTCAAGTACATCTGT-3' | 5'-GACGATTCGATTGGCAGGGAT-3' |
| β-actin. | 5′-TTCAACACCCCAGCCATG-3′ | 5′-CCTCGTAGATGG GCACAGT-3’ |

**Supplementary 2** **Fig. 1**： The correlation between the expression of all the S-palmitoylases and *S*-depalmitoylases and the pathological stage. *ZDHHC2*1 changed as the tumor progressed that it decreased in stage I to stage II. (*P*<0.05).


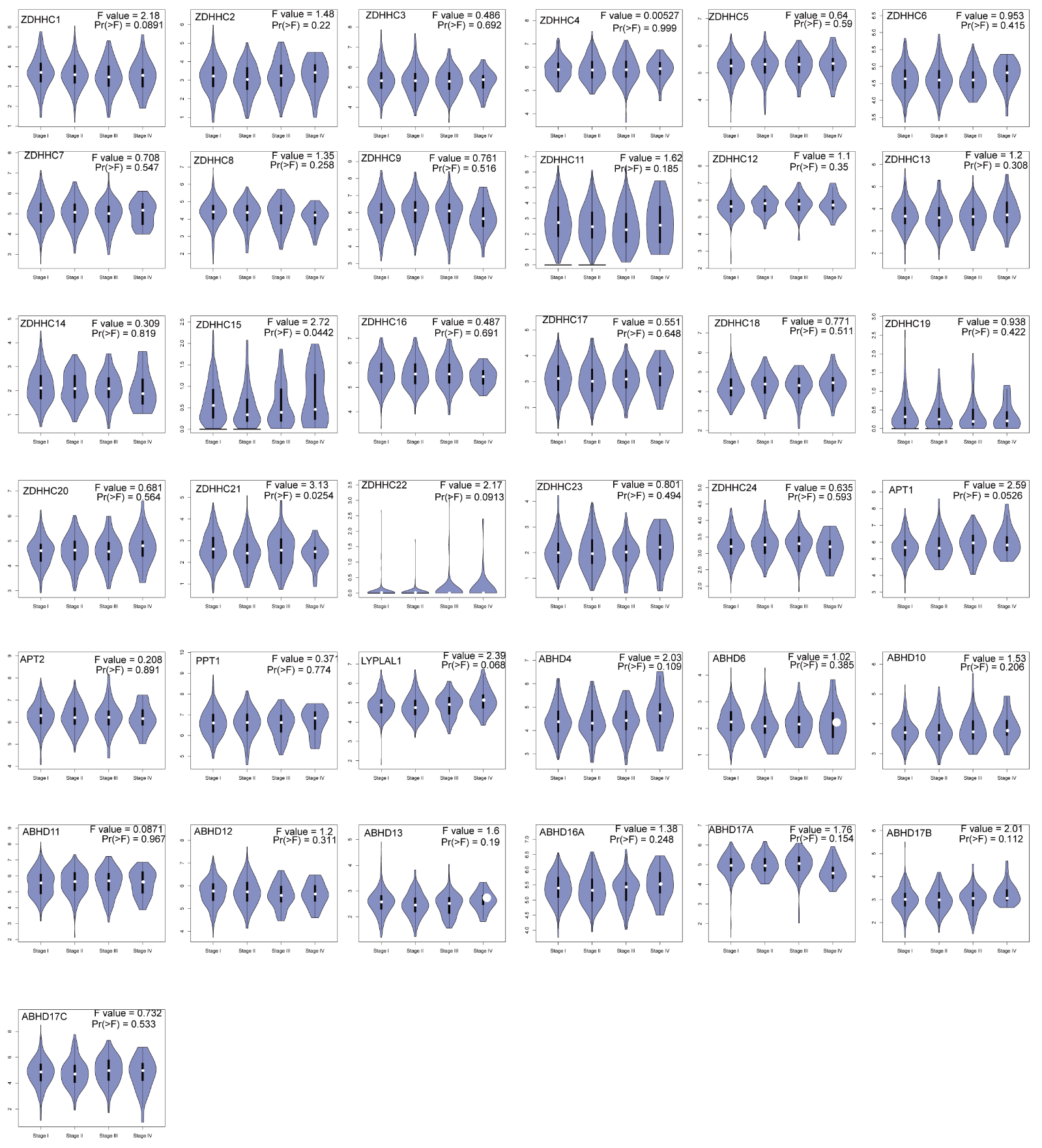


**Supplementary** 3 **Fig.2**： Functional and pathway enrichment analyses of S-palmitoylases and S-depalmitoylases were performed using METASCAPE and visualized in bar chart.


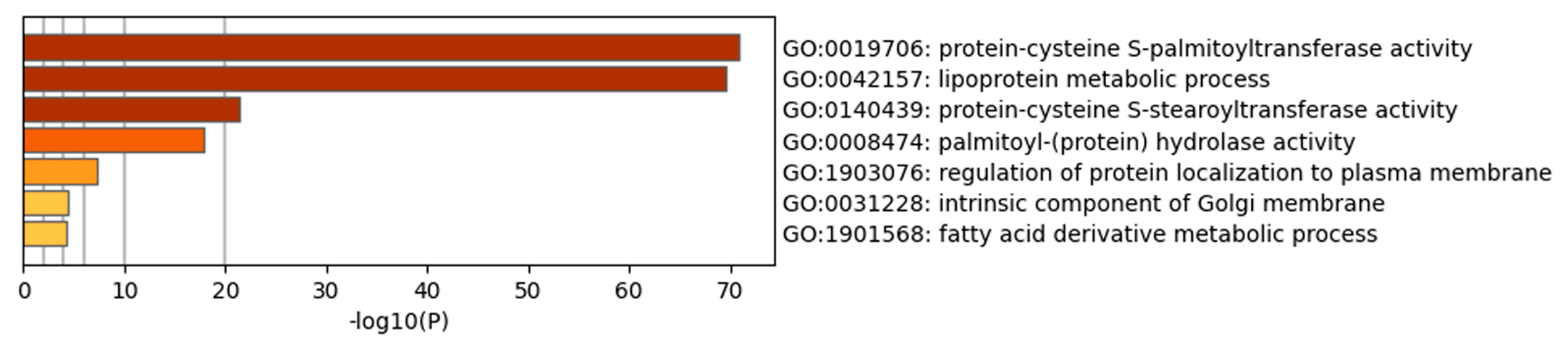


**Supplementary** **4** **Fig.3**： Kaplan-meier plot revealing the correlation between S-palmitoylases and S-depalmitoylases level and first progression survival in LUAD patients. Patients were divided into two groups including the high (the red line) and low (the black line) groups expression based on expression level.


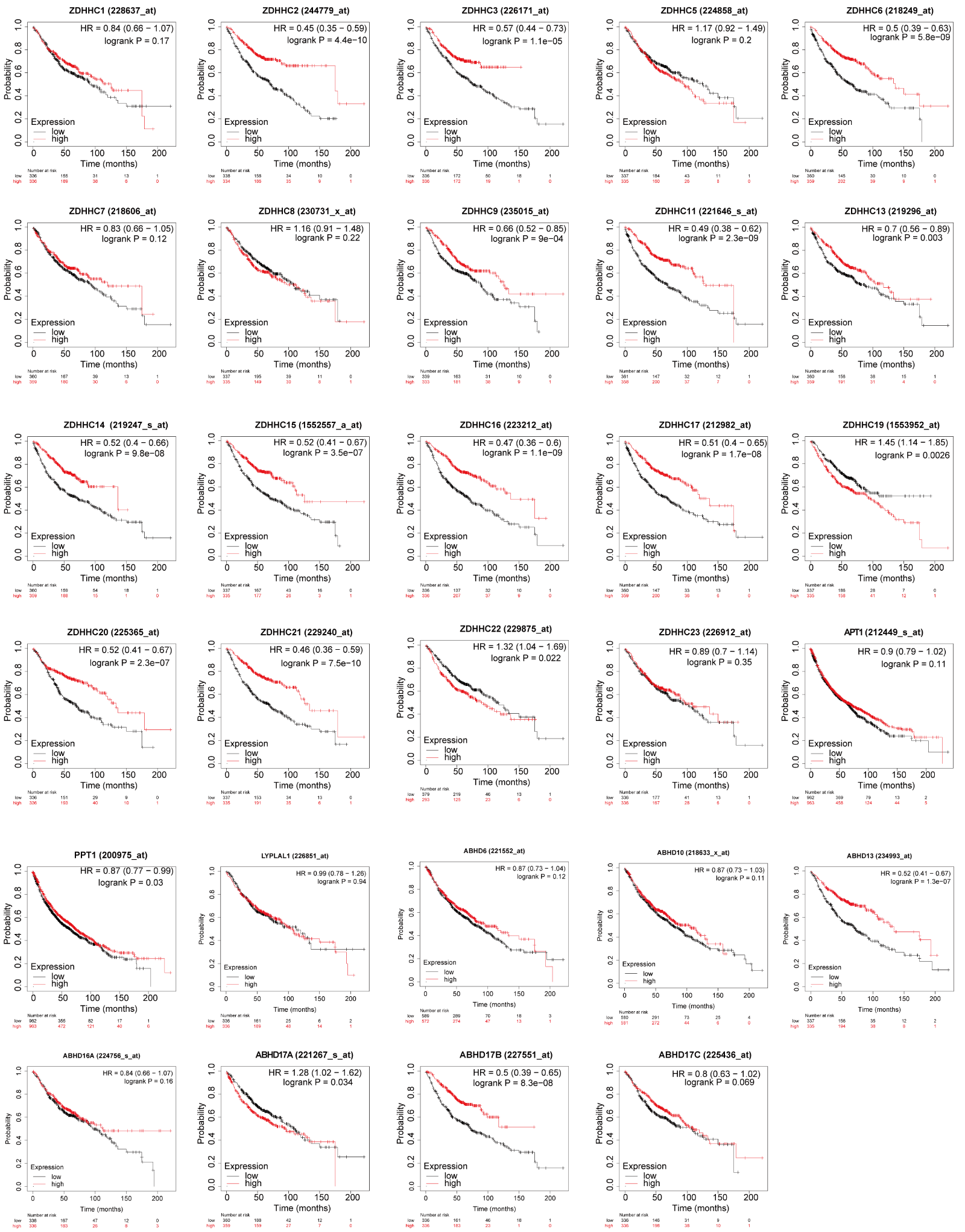


**Supplementary** 5 **Fig.4**： Kaplan-meier plot revealing the correlation between S-palmitoylases and S-depalmitoylases level and first progression survival in LUAD patients. Patients were divided into two groups including the high (the red line) and low (the black line) groups expression based on expression level.


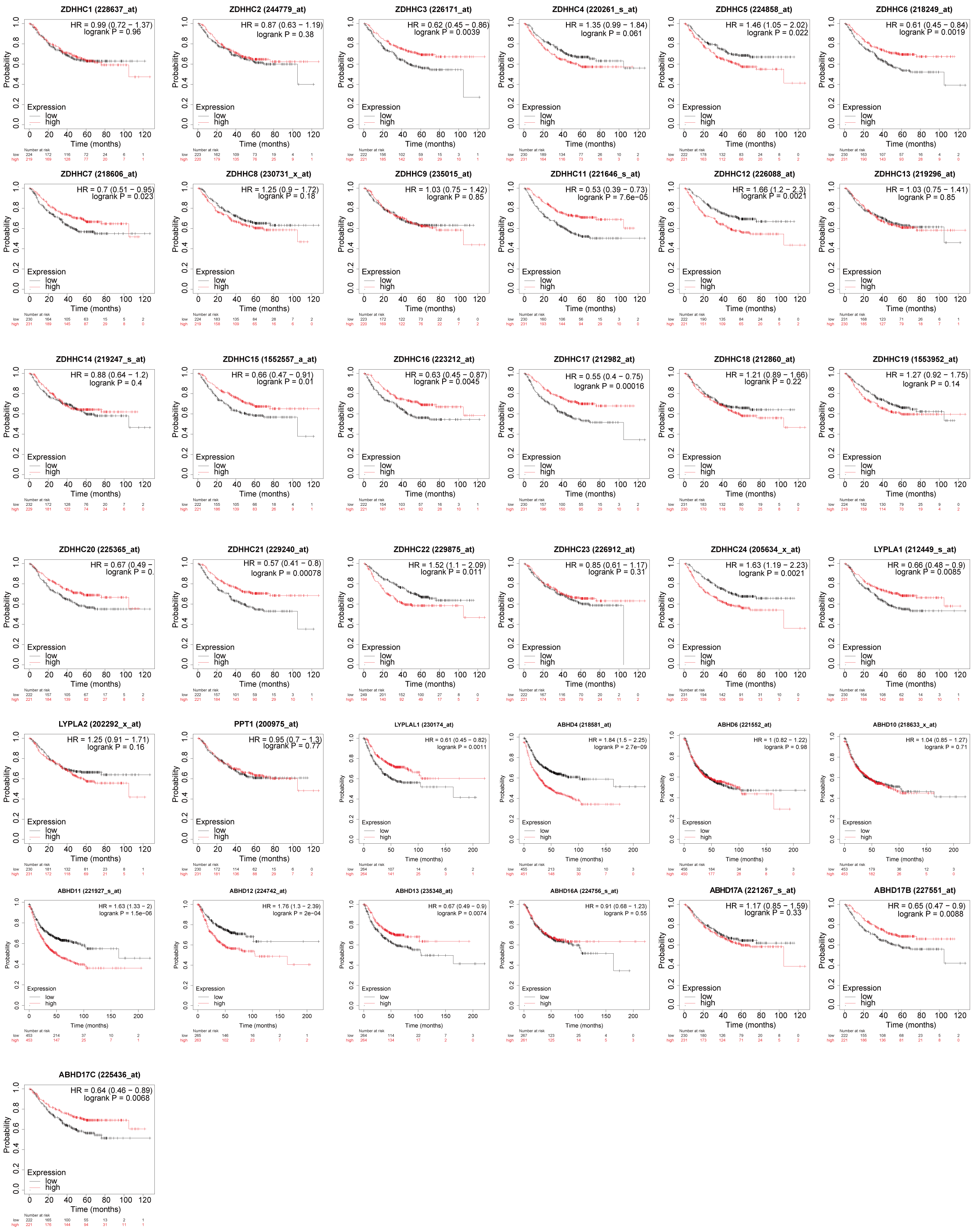


**Supplementary 6 Fig.5**： Kaplan-Meier plot revealing the correlation between S-palmitoylases and S-depalmitoylases level and post progression survival in LUAD patients. Patients were divided into two groups including the high (the red line) and low (the black line) groups expression based on expression level.


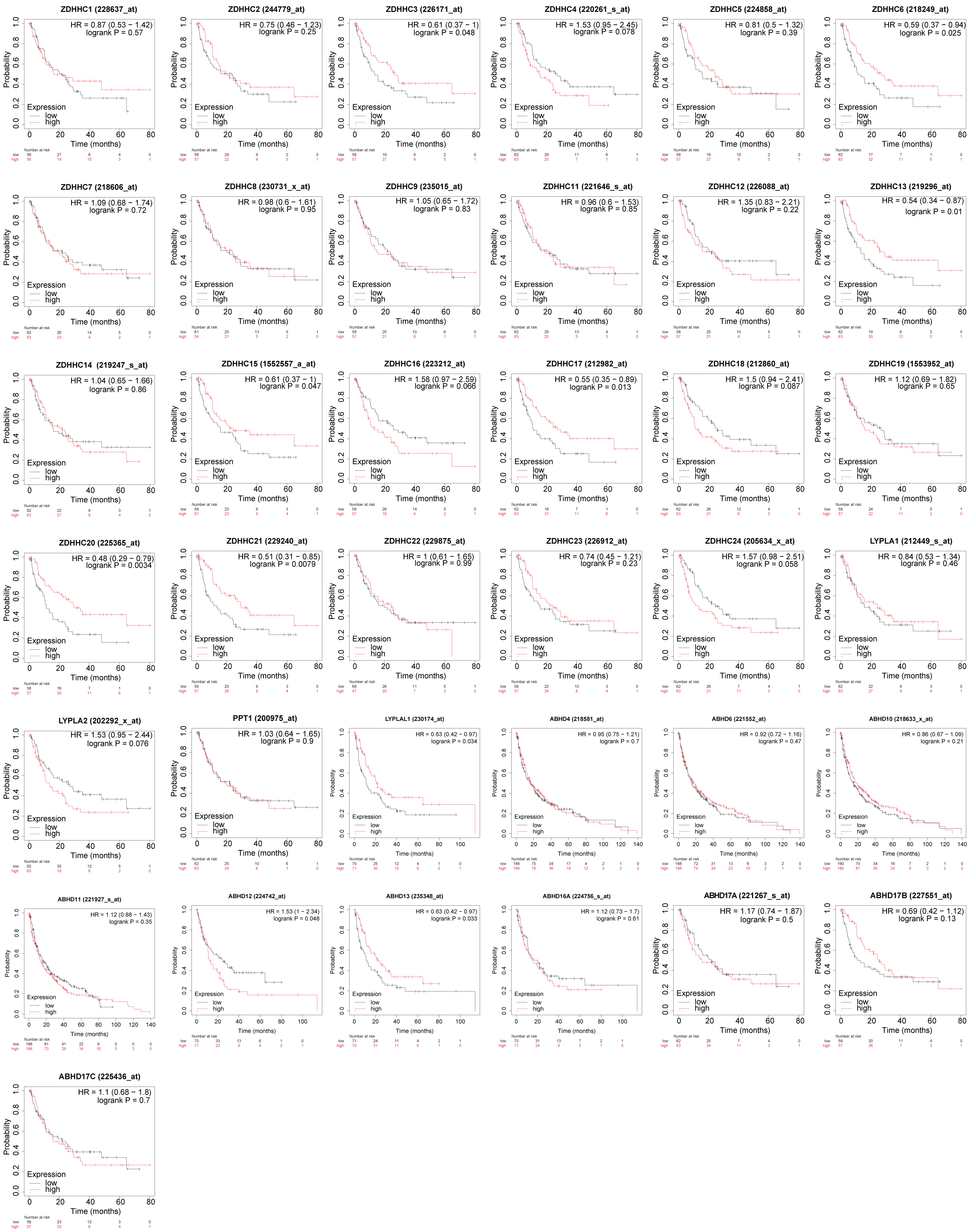


**Supplementary 7** **TABLE 2**: Relationships between ZDHHC4 mRNA expression and clinical characteristics in TCGA cohort (LUAD).

| Characteristic | Low expression of ZDHHC4 | High expression of ZDHHC4 | p |
| --- | --- | --- | --- |
| n | 267 | 268 |  |
| Sex, n (%) |  |  | 0.052 |
| Female | 131 (24.5%) | 155 (29%) |  |
| Male | 136 (25.4%) | 113 (21.1%) |  |
| Age, n (%) |  |  | 1.000 |
| <=65 | 127 (24.6%) | 128 (24.8%) |  |
| >65 | 130 (25.2%) | 131 (25.4%) |  |
| **Smoker, n (%)** |  |  | **0.001** |
| No | 24 (4.6%) | 51 (9.8%) |  |
| Yes | 235 (45.1%) | 211 (40.5%) |  |
| OS event, n (%) |  |  | 0.093 |
| Alive | 181 (33.8%) | 162 (30.3%) |  |
| Dead | 86 (16.1%) | 106 (19.8%) |  |
| T stage, n (%) |  |  | 0.099 |
| T1 | 94 (17.7%) | 81 (15.2%) |  |
| T2 | 134 (25.2%) | 155 (29.1%) |  |
| T3 | 30 (5.6%) | 19 (3.6%) |  |
| T4 | 7 (1.3%) | 12 (2.3%) |  |
| **N stage, n (%)** |  |  | **0.021** |
| N0 | 188 (36.2%) | 160 (30.8%) |  |
| N1 | 39 (7.5%) | 56 (10.8%) |  |
| N2 | 31 (6%) | 43 (8.3%) |  |
| N3 | 0 (0%) | 2 (0.4%) |  |
| M stage, n (%) |  |  | 0.292 |
| M0 | 177 (45.9%) | 184 (47.7%) |  |
| M1 | 9 (2.3%) | 16 (4.1%) |  |
| Pathologic stage, n (%) |  |  | 0.345 |
| Stage I | 156 (29.6%) | 138 (26.2%) |  |
| Stage II | 60 (11.4%) | 63 (12%) |  |
| Stage III | 38 (7.2%) | 46 (8.7%) |  |
| Stage IV | 10 (1.9%) | 16 (3%) |  |

**Supplementary 8** **TABLE 3**: Relationships between ZDHHC12 mRNA expression and clinical characteristics in TCGA cohort (LUAD).

| Characteristic | Low expression of ZDHHC12 | High expression of ZDHHC12 | p |
| --- | --- | --- | --- |
| n | 267 | 268 |  |
| Sex, n (%) |  |  | 0.514 |
| Female | 147 (27.5%) | 139 (26%) |  |
| Male | 120 (22.4%) | 129 (24.1%) |  |
| Age, n (%) |  |  | 0.379 |
| <=65 | 122 (23.6%) | 133 (25.8%) |  |
| >65 | 136 (26.4%) | 125 (24.2%) |  |
| Smoker, n (%) |  |  | 0.443 |
| No | 41 (7.9%) | 34 (6.5%) |  |
| Yes | 219 (42%) | 227 (43.6%) |  |
| OS event, n (%) |  |  | 0.134 |
| Alive | 180 (33.6%) | 163 (30.5%) |  |
| Dead | 87 (16.3%) | 105 (19.6%) |  |
| T stage, n (%) |  |  | 0.162 |
| T1 | 97 (18.2%) | 78 (14.7%) |  |
| T2 | 138 (25.9%) | 151 (28.4%) |  |
| T3 | 19 (3.6%) | 30 (5.6%) |  |
| T4 | 10 (1.9%) | 9 (1.7%) |  |
| **N stage, n (%)** |  |  | **0.017** |
| N0 | 185 (35.6%) | 163 (31.4%) |  |
| N1 | 37 (7.1%) | 58 (11.2%) |  |
| N2 | 31 (6%) | 43 (8.3%) |  |
| N3 | 0 (0%) | 2 (0.4%) |  |
| M stage, n (%) |  |  | 0.780 |
| M0 | 177 (45.9%) | 184 (47.7%) |  |
| M1 | 11 (2.8%) | 14 (3.6%) |  |
| **Pathologic stage, n (%)** |  |  | **0.024** |
| Stage I | 164 (31.1%) | 130 (24.7%) |  |
| Stage II | 53 (10.1%) | 70 (13.3%) |  |
| Stage III | 34 (6.5%) | 50 (9.5%) |  |
| Stage IV | 12 (2.3%) | 14 (2.7%) |  |

**Supplementary 9** **TABLE 4**: Relationships between ZDHHC18 mRNA expression and clinical characteristics in TCGA cohort(LUAD).

| Characteristic | Low expression of ZDHHC18 | High expression of ZDHHC18 | p |
| --- | --- | --- | --- |
| n | 267 | 268 |  |
| Sex, n (%) |  |  | 0.280 |
| Female | 136 (25.4%) | 150 (28%) |  |
| Male | 131 (24.5%) | 118 (22.1%) |  |
| Age, n (%) |  |  | 0.064 |
| <=65 | 115 (22.3%) | 140 (27.1%) |  |
| >65 | 140 (27.1%) | 121 (23.4%) |  |
| Smoker, n (%) |  |  | 0.930 |
| No | 36 (6.9%) | 39 (7.5%) |  |
| Yes | 220 (42.2%) | 226 (43.4%) |  |
| OS event, n (%) |  |  | 0.187 |
| Alive | 179 (33.5%) | 164 (30.7%) |  |
| Dead | 88 (16.4%) | 104 (19.4%) |  |
| T stage, n (%) |  |  | 0.810 |
| T1 | 90 (16.9%) | 85 (16%) |  |
| T2 | 141 (26.5%) | 148 (27.8%) |  |
| T3 | 26 (4.9%) | 23 (4.3%) |  |
| T4 | 8 (1.5%) | 11 (2.1%) |  |
| **N stage, n (%)** |  |  | **< 0.001** |
| N0 | 196 (37.8%) | 152 (29.3%) |  |
| N1 | 35 (6.7%) | 60 (11.6%) |  |
| N2 | 25 (4.8%) | 49 (9.4%) |  |
| N3 | 0 (0%) | 2 (0.4%) |  |
| M stage, n (%) |  |  | 0.195 |
| M0 | 186 (48.2%) | 175 (45.3%) |  |
| M1 | 9 (2.3%) | 16 (4.1%) |  |
| **Pathologic stage, n (%)** |  |  | **0.003** |
| Stage I | 166 (31.5%) | 128 (24.3%) |  |
| Stage II | 53 (10.1%) | 70 (13.3%) |  |
| Stage III | 31 (5.9%) | 53 (10.1%) |  |
| Stage IV | 10 (1.9%) | 16 (3%) |  |

**Supplementary** **10** **TABLE 5** ：Relationships between ZDHHC24 mRNA expression and clinical characteristics in TCGA cohort(LUAD).

| Characteristic | Low expression of ZDHHC24 | High expression of ZDHHC24 | p |
| --- | --- | --- | --- |
| n | 267 | 268 |  |
| **Sex, n (%)** |  |  | **0.034** |
| Female | 130 (24.3%) | 156 (29.2%) |  |
| Male | 137 (25.6%) | 112 (20.9%) |  |
| Age, n (%) |  |  | 0.159 |
| <=65 | 120 (23.3%) | 135 (26.2%) |  |
| >65 | 140 (27.1%) | 121 (23.4%) |  |
| Smoker, n (%) |  |  | 0.232 |
| No | 32 (6.1%) | 43 (8.3%) |  |
| Yes | 227 (43.6%) | 219 (42%) |  |
| OS event, n (%) |  |  | 0.629 |
| Alive | 168 (31.4%) | 175 (32.7%) |  |
| Dead | 99 (18.5%) | 93 (17.4%) |  |
| T stage, n (%) |  |  | 0.353 |
| T1 | 94 (17.7%) | 81 (15.2%) |  |
| T2 | 141 (26.5%) | 148 (27.8%) |  |
| T3 | 20 (3.8%) | 29 (5.5%) |  |
| T4 | 11 (2.1%) | 8 (1.5%) |  |
| **N stage, n (%)** |  |  | **0.020** |
| N0 | 185 (35.6%) | 163 (31.4%) |  |
| N1 | 44 (8.5%) | 51 (9.8%) |  |
| N2 | 27 (5.2%) | 47 (9.1%) |  |
| N3 | 0 (0%) | 2 (0.4%) |  |
| M stage, n (%) |  |  | 0.350 |
| M0 | 174 (45.1%) | 187 (48.4%) |  |
| M1 | 15 (3.9%) | 10 (2.6%) |  |
| **Pathologic stage, n (%)** |  |  | **0.008** |
| Stage I | 159 (30.2%) | 135 (25.6%) |  |
| Stage II | 61 (11.6%) | 62 (11.8%) |  |
| Stage III | 28 (5.3%) | 56 (10.6%) |  |
| Stage IV | 15 (2.8%) | 11 (2.1%) |  |

**Supplementary 11** **TABLE 6**： Relationships between LYPLA2 mRNA expression and clinical characteristics in TCGA cohort (LUAD).

| Characteristic | Low expression of LYPLA2 | High expression of LYPLA2 | p |
| --- | --- | --- | --- |
| n | 267 | 268 |  |
| **Sex, n (%)** |  |  | **0.034** |
| Female | 130 (24.3%) | 156 (29.2%) |  |
| Male | 137 (25.6%) | 112 (20.9%) |  |
| Age, n (%) |  |  | 0.426 |
| <=65 | 121 (23.4%) | 134 (26%) |  |
| >65 | 134 (26%) | 127 (24.6%) |  |
| Smoker, n (%) |  |  | 0.262 |
| No | 32 (6.1%) | 43 (8.3%) |  |
| Yes | 225 (43.2%) | 221 (42.4%) |  |
| OS event, n (%) |  |  | 0.812 |
| Alive | 173 (32.3%) | 170 (31.8%) |  |
| Dead | 94 (17.6%) | 98 (18.3%) |  |
| T stage, n (%) |  |  | 0.732 |
| T1 | 84 (15.8%) | 91 (17.1%) |  |
| T2 | 143 (26.9%) | 146 (27.4%) |  |
| T3 | 27 (5.1%) | 22 (4.1%) |  |
| T4 | 11 (2.1%) | 8 (1.5%) |  |
| N stage, n (%) |  |  | 0.389 |
| N0 | 175 (33.7%) | 173 (33.3%) |  |
| N1 | 48 (9.2%) | 47 (9.1%) |  |
| N2 | 32 (6.2%) | 42 (8.1%) |  |
| N3 | 2 (0.4%) | 0 (0%) |  |
| M stage, n (%) |  |  | 1.000 |
| M0 | 183 (47.4%) | 178 (46.1%) |  |
| M1 | 13 (3.4%) | 12 (3.1%) |  |
| Pathologic stage, n (%) |  |  | 0.869 |
| Stage I | 143 (27.1%) | 151 (28.7%) |  |
| Stage II | 63 (12%) | 60 (11.4%) |  |
| Stage III | 39 (7.4%) | 45 (8.5%) |  |
| Stage IV | 14 (2.7%) | 12 (2.3%) |  |

**Supplementary 12** **Fig.6**： Genetic alteration in LUAD patients of S-palmitoylases and S-depalmitoylases in LUAD.


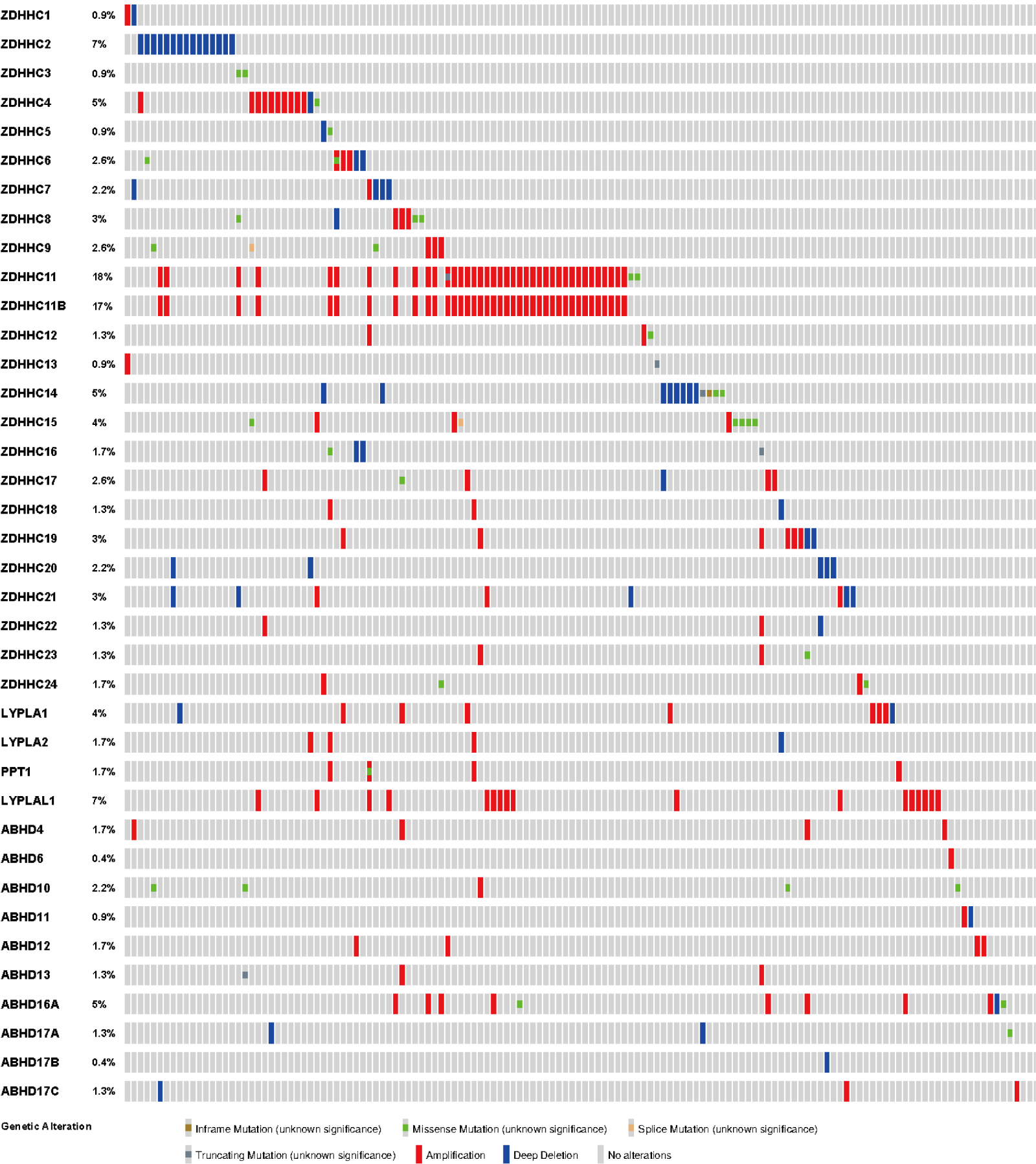


**Supplementary 13** **Fig.7** Immunohistochemistry images of ZDHHC4/12/18/24 and ABHD4/11/12 in LUAD tissues and healthy control tissues (Human Protein Atlas).

*
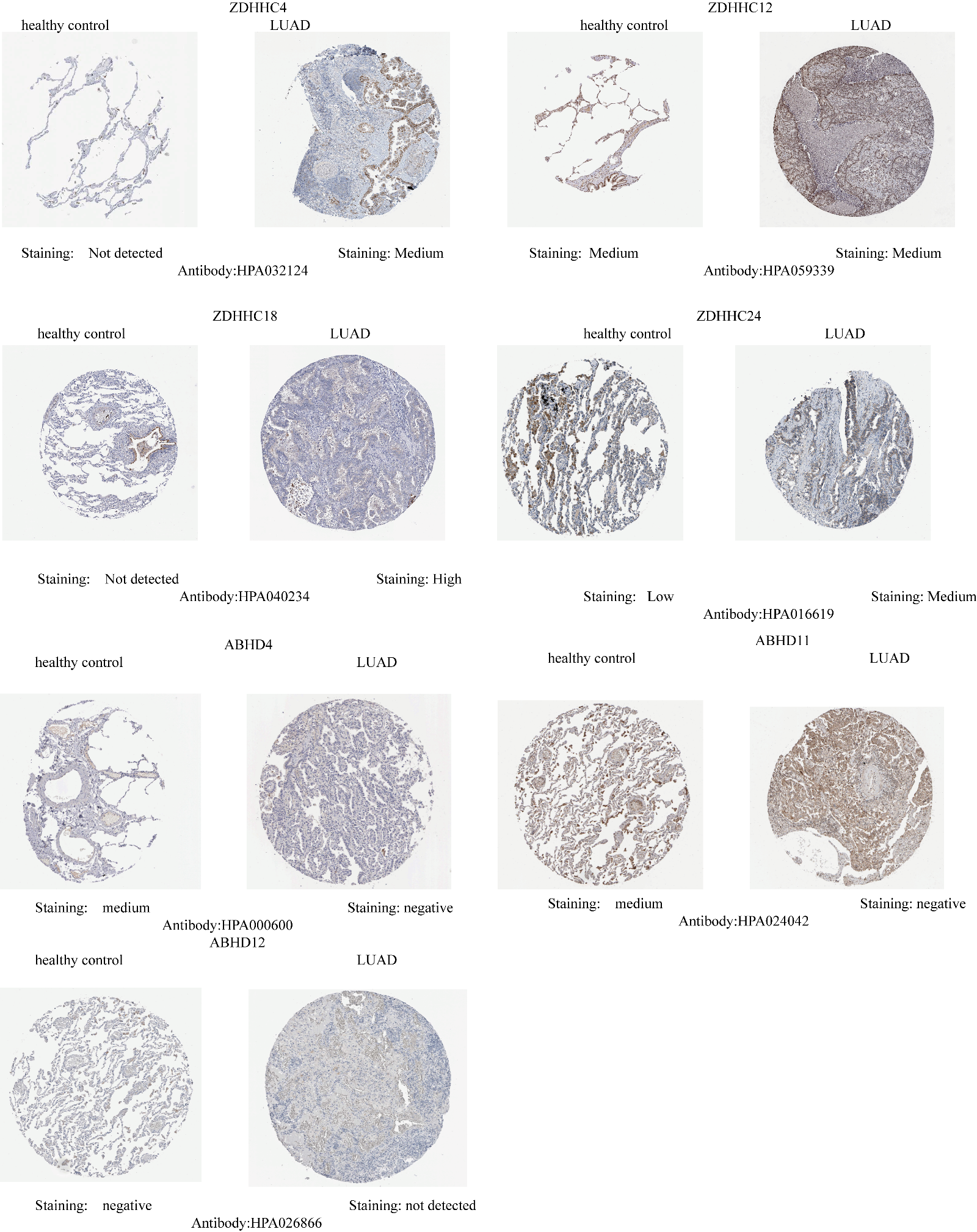
*

**Supplementary 14 Table 7**： Immune checkpoint-related gene CD276 palmitoylation sites.

| **predicted sites** | |  |  |  |
| --- | --- | --- | --- | --- |
| **ID** | **Position** | **Peptide** | **Score** | **Cutoff** |
| **CD276** | **220** | VVLGANGTYSCLVRNPVLQQD | **0.8976** | **0.892** |
| **CD276** | **438** | VVLGANGTYSCLVRNPVLQQD | **0.8976** | **0.892** |
| **CD276** | **488** | IALLVALAFVCWRKIKQSCEE | **0.9716** | **0.892** |
